# Psychological and cognitive outcomes in patients with unruptured intracranial aneurysms and aneurysmal subarachnoid haemorrhage: a multidimensional assessment

**DOI:** 10.1007/s00701-025-06732-z

**Published:** 2025-12-01

**Authors:** Vera Beallo, Tamas Nemeth, Pal Barzo, Mona Stankovic

**Affiliations:** 1 Department of Neurosurgery, Albert Szent-Györgyi Medical School, University of Szeged, 6 Semmelweis Utca, Szeged, 6725 Hungary; 2 Doctoral School of Clinical Medicine, Albert Szent-Györgyi Medical School, University of Szeged, 6 Korányi Fasor, Szeged, 6720 Hungary

**Keywords:** Intracranial aneurysm, Subarachnoid haemorrhage, Psychological outcomes, Quality of life, Cognitive assessment, Affective symptoms

## Abstract

**Introduction:**

Unruptured intracranial aneurysms (UIAs) and aneurysmal subarachnoid hemorrhage (aSAH) pose not only neurological but also psychological and cognitive challenges. This study aimed to compare patients with UIA and aSAH and explore how psychological symptoms relate to their quality of life (QoL).

**Methods:**

Between May 2023 and May 2025, 128 patients (aged 31–79, *M* = 53.9, *SD* = 7.24) were enrolled: 63 with aSAH and 65 with UIA. All aSAH patients had favourable neurological outcomes (modified Rankin Scale score of 0 or 1). Assessment included the Montreal Cognitive Assessment (MoCA), State-Trait Anxiety Inventory – Trait (STAI-T), Beck Depression Inventory – Short Form (BDI-SF), Athens Insomnia Scale (AIS), Toronto Alexithymia Scale (TAS-20), and WHOQOL-BREF.

**Results:**

There were no significant psychological or cognitive differences between the aSAH and UIA groups; thus, they were analysed together. Compared to normative data, patients showed reduced cognitive performance and elevated depressive symptoms. Psychological well-being was the most affected QoL domain. All psychological variables negatively correlated with QoL, with anxiety, depression, and sleep disturbances emerging as significant predictors in linear regression.

**Discussion:**

Our results suggest that emotional and sleep-related symptoms have a more substantial impact on QoL than cognitive impairment. The lack of group differences implies that psychological vulnerability may already be present before aneurysm rupture, underlining the need for early intervention.

**Conclusion:**

The knowledge of having an aneurysm alone places a significant psychological burden on patients even without SAH, affecting cognitive and psychological well-being and quality of life. Therefore, psychological follow-up and care of patients diagnosed with intracranial aneurysms should be a priority.

**Trial registration:**

Not applicable because the health-related intervention was not conducted in this study.

## Introduction

Intracranial aneurysms — particularly unruptured intracranial aneurysms (UIAs) and those associated with aneurysmal subarachnoid hemorrhage (aSAH) — pose significant clinical challenges not only in terms of neurological outcomes but also in terms of their psychological and cognitive consequences. These conditions are frequently associated with elevated levels of anxiety, depression, cognitive deficit and a decreased quality of life [1, 6, 9, 11, 13].

UIAs are relatively prevalent and many cases have been incidentally identified during neuroimaging for unrelated reasons. Knowledge of aneurysms causes significant distress to patients after diagnosis, mainly due to persistent fear of rupture — frequently described as living with a “ticking time bomb” [10]. The anxiety this causes significantly reduces patients' quality of life, even without bleeding occurring [10, 11]. Although the direct cognitive consequences of UIAs remain underexplored, emerging evidence suggests that chronic stress related to fear of rupture could indirectly impair cognitive function [10]. This underscores the importance of incorporating comprehensive psychological assessment and support into the care of patients diagnosed with UIAs, as mental health status can significantly influence overall well-being and treatment decisions [13].

The consequences of SAH resulting from aneurysm rupture are much better known. Cognitive impairment is a common sequela, affecting an estimated 7% to 60% of survivors, and may persist long after the acute phase [17]. Deficits frequently involve memory, executive function, and processing speed, which contribute to significant challenges in daily activities and overall health-related quality of life [1]. Furthermore, patients often experience difficulties in social cognition and emotional regulation: approximately one-third of patients exhibited impaired interpersonal behaviour, apathy, and challenges in self-awareness, which may link to emotional processing disorders, including alexithymia [6]. Alexithymia is a cognitive-affective processing deficit that is often associated with psychological and somatic disorders. In this condition, patients have difficulty identifying and expressing their own emotions, find it difficult to separate emotions from bodily sensations, and tend to focus their thinking on external events and have poor imagination [31]. These cognitive difficulties are often compounded by psychological trauma related to the hemorrhagic event. Elevated levels of anxiety and depression have been documented, driven both by the traumatic nature of the event and by uncertainty about future health [1, 13, 17]. These mental health concerns also influence sleep quality in this population. Higher levels of anxiety and depression are associated with sleep disturbances [9]. Complex cognitive and affective problems, significant psychological distress due to functional outcomes and the traumatic nature of bleeding, as well as deterioration in sleep quality, all have an impact on patients' well-being and subjective quality of life [1, 7, 13, 33].

The relationship between cognitive-psychological outcomes and the treatment modality is complex. For instance, the choice between surgical clipping and endovascular techniques may influence postoperative cognitive trajectories, with some evidence indicating that minimally invasive approaches may be more protective of cognitive functions [35]. In Hungary, in accordance with international and national professional guidelines, ruptured aneurysms are primarily treated endovascularly; if this is not technically feasible, the procedure is performed surgically (with clipping). In the case of unruptured aneurysms, an individual risk assessment is always performed. During this assessment, we take into account the PHASES score, but the opinion of the physician regularly involved in the treatment of aneurysm patients (neurosurgeon and neurointerventional specialist) is at least as important. The morphology of the aneurysm and an individual assessment of its location also play a role in the decision. Treatment is generally considered for aneurysms larger than 3 mm, especially if they are irregularly shaped, lobulated, or show growth and/or change during follow-up. In terms of aneurysm location, for example, aneurysms located in the anterior communicating artery bleed more frequently, so we are more likely to decide on treatment for these. The decision also takes into account the patient's age, general condition, comorbidities, and the expected risks and long-term benefits of the procedure. The treatment decision is always made by a multidisciplinary team (neurosurgeon, neurointerventional specialist) together with the patient, after providing detailed information. In Hungary, aneurysm treatments are fully funded by the National Health Insurance Fund (NEAK) within the framework of the public health care system, free of charge for patients. Doctors and healthcare institutions are covered by state funding and do not receive any financial incentives or extra allowances for individual procedures. Treatment decisions are therefore based solely on medical and patient safety considerations and are not influenced by economic considerations. Given the intricate interplay among physical, cognitive, and psychological factors in patients with UIAs and aSAH, a comprehensive multidisciplinary approach is essential. Management strategies should extend beyond immediate neurological care to include long-term psychological and cognitive support.

The aim of the present study is to compare patients with UIAs and those with aSAH in terms of anxiety, depression, sleep quality, and cognitive outcomes, and to explore how the onset of psychological symptoms affects their quality of life. In addition, we would like to compare patients with ruptured and unruptured aneurysms with a healthy sample to see how cognitive and psychological status and quality of life change after aneurysm diagnosis. Our goal is to gain a more accurate understanding of the' condition and needs of our patients, enabling us to support their recovery holistically — attending not only to physical symptoms, but also to the cognitive and psychological dimensions of their experience.

## Methods

### Patient inclusion

The research participants were divided into two groups. The study group consisted of patients who had undergone an aneurysmal subarachnoid hemorrhage (aSAH), while the control group consisted of patients with confirmed unruptured intracranial aneurysms (UIAs). Criteria for inclusion in the study were: for both groups, the patient had to be at least 18 years of age, for the aSAH group I. confirmed subarachnoid haemorrhage, II. diagnosis of one aneurysm (we did not follow up on the results of further angiographic examinations, which could have confirmed multiple aneurysms), III. at least six months had elapsed since the haemorrhage, IV. patient's condition had improved to the extent that he/she could be included in the co-operative study (cognitive function was not impaired to the extent that inclusion in the psychological study was meaningless), V. no ischaemic lesions in eloquent area of the brain on control imaging studies, VI. modified Rankin Scale (mRS) < 2, i.e. a patient who is completely asymptomatic or mildly symptomatic and self-caring according to the scale. The second group included patients who I. had one confirmed unruptured intracranial aneurysm (we did not follow up on the results of further angiographic examinations, which could have confirmed multiple aneurysms), II. had been treated (clipping or coiling) or followed up in the clinic for the aneurysm. The distribution of treatment methods for aSAH and UIA patients is detailed in Table 1. In this study, the location of the aneurysm was not taken into account in either group. For both groups, the exclusion criterion was any prior psychiatric or neurological diagnosis in order to exclude factors that have a greater impact on cognitive and psychological status than the diagnosis of aneurysm or SAH. In both pathologies, there are frequently occurring comorbidities which, if left untreated, can affect cognitive and psychological status through their complications, such as hypertension (HT) and diabetes mellitus (DM). Patients whose comorbidities are being treated may have been included in the sample, as we believe that monitored and medically controlled comorbidities do not significantly affect our results.

**Table 1:** The characteristics of aSAH and UIA patients at the time of the examination

		aSAH patients	UIA patients
Number		63	65
Mean age (*SD*)		53.9 (7.24)	56.1 (9.84)
Minimum–maximum age		34–70	31–79
Sex	Female (%)	46 (73%)	54 (83%)
	Male (%)	17 (27%)	11 (17%)
Mean years of education (*SD*)		12.2 (3.63)	13.9 (4.31)
Average years since diagnosis		4.25	2.34
Minimum and maximum number of years since diagnosis		0.5–18	0.5–14
Treatment method number of patients (%)	Not treated (follow up)	-	15 (23%)
	Clipping	6 (9.5%)	1 (1.5%)
	Coiling	57 (90.5%)	49 (75.5%)

### The examination process

This study was approved by the Scientific and Research Ethics Committee of the Medical Research Council of Hungary (IRB number BM/33100–0/2023) and the Regional Research Ethics Committee of the University of Szeged (IRB number 38/2023-SZTE RKEB). Patients were selected using the patient registration system (eMedSolution) used in the Albert Szent-Györgyi Clinical Center. All patients were individually interviewed by a psychologist one time, which allowed psychological intervention where necessary. All of the interviews were conducted at the Department of Neurosurgery, University of Szeged, Szent-Györgyi Albert Clinical Centre. Since we did not have our own healthy sample in addition to the aSAH and UIA patients, we based our statistical analyses on the national standard values measured on a healthy Hungarian sample using the cognitive test and psychological questionnaires validated and standardized in Hungarian, as detailed below [12, 19, 20, 22, 25, 27].

### Assessments

#### Montreal cognitive assessment (MoCa)

To detect cognitive impairment, we used the Montreal Cognitive Assessment (MoCa) [18, 32]. Our choice was guided by the fact that although there is no international consensus-based neuropsychological screening routine for the aSAH and UIA population, MoCa has been found to be an appropriate measure of sensitivity for this purpose [24, 34, 36]. The assessment tests orientation, spatial-visual, memory, language, attention, abstraction, and executive functions. The test can be scored up to 30 points as the sum of the scores on each sub-task (plus one point for less than 12 years of schooling), with a Hungarian clinical cut-off of 24 points for mild cognitive impairment and 17 points for dementia. This is a stricter cut-off than in the original version of the test, where the clinical cut-off is 26 points.

#### Spielberger state and trait anxiety questionnaire (STAI-S/T)

Anxiety severity was assessed with the Spielberger State and Trait Anxiety Questionnaire (STAI-S/T), which consists of 40 items, 20 of which relate to state anxiety, which maps the current, subjectively experienced sense of fear, increased autonomic nervous system functioning, i.e., a kind of temporary state of tension [27, 30]. In contrast, the 20 items for trait anxiety examine a personality trait, and therefore a more permanent state, the individual's tendency to become anxious. In the analyses, we considered the trait anxiety values, which is a more stable indicator of the condition.

#### Beck depression questionnaire (BDI-SF)

Depression was measured using a sort version of the Beck Depression Questionnaire (BDI-SF) [5, 14, 26]. The questionnaire consists of 9 items that measure the severity of depressive symptoms. The scores are used to classify the respondent's depressive state: 0–9 is normal, 10–18 is mild depression, 19–25 is moderate depression, and above 25 is severe depression.

#### Toronto alexithymia scale (TAS-20)

To assess alexithymia, we chose the most commonly used Toronto Alexithymia Scale (TAS-20) [2, 3, 8]. The researchers who developed the test divided alexithymia as a symptom into three components: difficulty in recognizing emotions, difficulty in naming them, and an outward-oriented thinking style, i.e., the person focuses on external events rather than internal experiences. The total score of the questionnaire is 100 points, and the clinical cut-off point is 61 points, above which the respondent is considered to have alexithymia.

#### Athens insomnia scale (AIS)

To assess sleep quality, we used the Athens Insomnia Scale (AIS) questionnaire, which is used to evaluate sleep disorders and diagnose insomnia. It consists of 8 items, of which 5 are about symptoms occurring at night and 3 are about the daytime consequences of these symptoms [20, 28, 29]. If the score is higher than 10 points, the patient suffers from insomnia.

#### World health organization (WHO) short quality of life questionnaire (WHOQOL-BREF)

To assess quality of life, we used the World Health Organization (WHO) abbreviated quality of life questionnaire (WHOQOL-BREF) [21]. The Hungarian version of the questionnaire is suitable for characterizing the health status and quality of life of the groups of patients. It consists of a total of 26 questions, after 2 general questions (global quality of life and health), the items are divided into 4 domains: 1. physical condition 2. psychological condition 3. social relations 4. environment. The higher the score, the better the quality of life is considered.

## Results

All statistical analyzes were conducted with Jamovi version 2.3.28.0 (The jamovi project, 2022). Data were analyzed, without missing data or outliers, and subsequently no one was excluded from the sample. Between May 2023 and May 2025, a total of 128 patients participated in the study from 31 to 79 years, with an average age of 55 years (*SD* = 8.70). 63 patients who have undergone aSAH and 65 patients with UIA (Table 1).

According to our data, 30.4% of patients who suffered aSAH were unable to return to work at all after the hemorrhage, 11.6% returned to a position they considered significantly worse than the one they had before the hemorrhage, 46.6% returned to the same or a similar position, and 11.6% retired regardless of the hemorrhage. 63.2% of UIA patients are working, 19.1% are retired, and 17.6% were not working at the time of the study, but we have no further data on the exact reasons for leaving work in this group. With regard to hypertension (HT) and diabetes mellitus (DM) as comorbidities, 22 patients (35%) who had undergone aSAH had no comorbidities, 39 patients (62%) had HT, and 2 patients (3%) had HT and DM. Among UIA patients, 19 (29%) had no comorbidities, 41 (63%) had HT, and 5 (8%) had HT and DM. In all cases, comorbidities, HT, and DM were treated with medication, as reported by the patients during the examination. 63% of all patients admit to smoking, which is a cardiovascular risk factor.

First time we divided the participants into two groups: 63 patients with aSAH were included in the study group, while 65 patients with unruptured intracranial aneurysms were included in the control group. We did not divide the UIA group into further subgroups based on different treatment methods, as there was no significant difference between the different treatment methods in terms of the variables we examined (*p* > 0.152 in all cases) and the sample size of the subgroups thus formed would not be balanced, which would make it difficult to draw conclusions. The means of the two groups in the tests were compared using an independent samples t-test. Although the condition of normal distribution was not fulfilled (*Shapiro–Wilk p* < 0.001 in all cases), the sample size (*n* = 128) suggests that the distribution of sample means is close to normal according to the theorem of marginal central distribution, which justify the use of a t-test. The homogeneity of the variance condition was met in all cases (*Levene's p* > 0.187 in all cases). A comparison of the results of the two groups' in the tests using the t-test is shown in Table 2.

**Table 2:** Comparison of aSAH and UIA patients

Test	aSAH mean* (SD)*	UIA mean* (SD)*	*t*	*df*	*p*	*CI*	*Cohen’s d*
MoCa	23.8 (3.57)	24.5 (3.12)	−1.18	126	0.241	95% [−0.56*;*0.14]	−0.21
STAI-T	42.7 (14.6)	42.8 (15.1)	−0.05	126	0.960	95% [−0.36*;*0.34]	−0.01
BDI-SF	13.3 (5.52)	12.9 (5.93)	0.36	126	0.721	95% [−0.28*;*0.41]	0.06
TAS-20	46.5 (15.8)	43.3 (14.0)	1.21	126	0.229	95% [−0.14*;*0.56]	0.24
AIS	5.03 (4.42)	5.05 (5.10)	−0.02	126	0.986	95% [−0.35*;*0.34]	−0.01

*n* = 128. aSAH group *n* = 63, UIA group *n* = 65

In the aSAH group, 25 (40%) of the patients scored below the clinical cut-off score of 24 points, suggesting mild cognitive impairment (MCI), and 4 (6%) scored below 17 points, suggesting dementia. Of the UIA group, 18 (28%) scored below 24 points and showed mild cognitive impairment, with no individuals in this group scoring below 17 points. In the aSAH group, 26 people (41%) were more distressed than the gender average, and 22 people (34%) in the UIA group. Of those who had experienced a hemorrhage, 36 people (57%) were classified as mildly depressed, 3 people (5%) as moderately depressed and 5 people (8%) as severely depressed on the BDI-SF scale. Of those with UIA, 37 (57%) were mildly depressed, 3 (5%) moderately depressed and 5 (8%) severely depressed. The two groups are also very similar for alexithymia, with 9 people (14%) in the aSAH group and 8 people (12%) in the UIA group scoring above the clinical cut-off and being considered as living with alexithymia. Sleep quality deterioration also shows similar results, with 8 people (13%) experiencing insomnia after aSAH and 10 people (15%) in the UIA group experiencing insomnia.

We have rejected our initial hypothesis according to which there is a significant difference between the groups in the above measured variables. Based on this result, we have combined the patients into one sample and compared their results with averages previously published and measured on a healthy control population [12, 19, 20, 22, 27]. The comparison was based on the results of Hungarian studies that investigated the mean score and standard deviation of a given test in a healthy sample. Table 3 shows the results of the one-sample t-tests compared to the reference values of the literature. The normal distribution of the data was also not met in these cases (*Shapiro–Wilk p* < 0.001 in all cases), the sample size (*n* = 128) again justifies the use of a t-test.

**Table 3:** Comparison of neurosurgical patients with a healthy sample

Test	Patient mean (*SD*)	Controll mean (*SD*) [12, 19, 20, 22, 27]	*t*	*df*	*p*	*CI*	*Cohen’s d*	
MoCa	24.2 (3.35)	26.4 (3.73)	−6.14	127	** < 0.001**	95% [−0.73*;*−0.36]		−0.54
STAI-T	42.7 (14.8)	42.4 (10.0)	0.56	127	0.575	95% [−0.12*;*0.22]		0.05
BDI-SF	13.1 (5.71)	4.84 (4.88)	18.0	127	** < 0.001**	95% [1.33*;1.85*]		1.59
TAS-20	44.9 (14.9)	43.2 (9.70)	1.41	127	0.161	95% [−0.05*;*0.53]		0.13
AIS	5.04 (4.42)	3.0	0.09	127	0.926	95% [−0.17*;*0.18]		0.01

The mean score of the AIS test control was determined by the mean age of the patient sample. The standard deviation for the age zone was not reported by the authors

From the above results it can be seen that patients with aSAH and UIA differ significantly from the healthy Hungarian average in cognitive performance and depression. The mean depression score of the patients (13.1 points) falls into the mild depression category according to the zonal classification of the BDI-SF. The mean MoCa score (24.2 points) is close to the clinical cut-off point (24 points) of the Hungarian version, which does not yet indicate mild cognitive impairment (MCI), however, the original version's cut-off point of 26 points places the sample mean in the MCI category, which is definitely an indication and should be taken into account when interpreting the results.

Quality of life aspects were assessed separately in patients with aSAH (*n* = 63) and UIA (*n* = 65). The statistics describing the quality of life of the two groups are shown in Table 4. It should be noted that the lowest mean score for the different aspects of quality of life was psychological well-being for both groups of patients, which highlights the psychological impact of aSAH and UIA.

**Table 4:** Different aspects of quality of life in patients with aSAH and UIA

	Group	*Mean*	*SD*	*Minimum*	*Maximum*
*Physical health*	aSAH	71.9	21.5	14	100
	UIA	73.6	24.4	0	100
*Psychological condition*	aSAH	41.1	13.5	0	58
	UIA	44.3	14.1	0	58
*Social contacts*	aSAH	72.7	21.6	17	100
	UIA	76.9	21.9	33	100
*Environment*	aSAH	82.4	12.8	44	100
	UIA	81.6	14.0	41	100
*Global quality of life and health*	aSAH	69.7	22.2	0	100
	UIA	63.1	23.1	0	100

aSAH group *n* = 63; UIA group *n* = 65

The presumed deterioration in quality of life compared to controls was assessed along the different domains offered by the questionnaire for the combined two groups of patients, as the mean scores for each domain did not differ significantly between aSAH and UIA patients in this case (*p* > 0.103 in all cases). Four domains were compared: physical health, psychological well-being, perception of social relationships, and environment. Patients were compared with the adult Hungarian mean [25]. A one-sample t-test was used for comparison. As before, the normal distribution of the data was not satisfied in these cases (*Shapiro–Wilk p* < 0.001 in all cases), but the sample size (*n* = 128) allowed for a parametric test in this case. The results are shown in Table 5.

**Table 5:** Comparison of different aspects of quality of life

	*Patient mean (SD)*	Control mean (*SD*)	*t*	*df*	*p*	*CI*	*Cohen’s d*
*Physical health*	72.8 (22.9)	58.44 (14.04)	7.31	127	** < 0.001**	95% [0.45*;0.84*]	0.65
*Psychological condition*	42.7 (13.9)	62.32 (15.62)	−15.70	127	** < 0.001**	95% [−1.63*;−1.15*]	−1.39
*Social contacts*	74.8 (21.8)	62.83 (16.83)	6.14	127	** < 0.001**	95% [1.17*;1.66*]	0.54
*Environment*	82.0 (13.4)	61.99 (15.09)	16.9	127	** < 0.001**	95% [0.36*;0.73*]	1.49

Group of pateints *n* = 128. Healthy control group *n* = 243

Subjective perceptions of quality of life differ significantly in all domains. However, of the four aspects, only psychological well-being shows a deterioration in patients with aSAH and UIA. The mean scores in the other domains are higher in this group of patients than in healthy controls. This was an expected result, as the mean age of the healthy adult study sample used for comparison was 70.2 years (*SD* = 7.1), which is significantly higher than the mean age of the patients, which was 55 years (*SD* = 8.70). However, it is surprising that the psychological state of the patients is significantly worse than the psychological state of a much older control group.

Pearson correlations were used to examine which psychological aspects are associated with quality of life. To use quality of life as a single indicator in our analyses, we averaged the transformed scores for each domain. It is important to note that quality of life is multidimensional, the different domains are separate but equally important domains that differ in content and item number, and the WHO recommends that they be interpreted separately. However, aggregation is essential for some statistical calculations, so for these we have taken advantage of this possibility (correlation analysis and linear regression). Cognitive functioning, anxiety, depression, alexithymia, and sleep quality also show moderate-strong significant correlations with quality of life (*p* < 0.001 in all cases). People who have better cognitive abilities, less anxiety and depression, better ability to recognize and express emotions, and better sleep are perceived to have a better quality of life. However, we cannot yet infer causality from this, only co-morbidity, so we further investigated which of these psychological factors predict quality of life. To explore predictor factors, we used multiple linear regression, whose prerequisites were met: linear relationship between predictors and the outcome variable, no multicollinearity (*VIF* < 2.97 for all variables; tolerance > 0.336), no autocorrelation (*DW* = 2.01), homogeneity of variance (*Shapiro–Wilk p* = 0.154), no outliers biasing model results (*Cook's D* < 0.5). The model is significant (*F*(5, 122) = 48.1, *p* < 0.001), explaining 66,3% of the variance in the population (*R*^*2*^ = 0.663 and adj. *R*^*2*^ = 0.649). The model for multiple linear regression is shown in Table 6.

**Table 6:** A linear regression model of quality-of-life predictors in patients with aSAH and UIA

Predictor	*B*	*β*	*SE*	*t*	*p*
Intercept	90.012		7.219	12.469	***p* < 0.001**
STAI-T	−0.519	−0.500	0.082	−6.210	***p* < 0.001**
AIS	−0.728	−0.229	0.243	−2.999	**0.003**
BDI-SF	−0.480	−0.182	0.240	−2.003	**0.047**
MoCa	0.356	0.079	0.248	1.439	0.153
TAS-20	0.020	0.019	0.080	0.243	0.808

*n* = 128. (*F*(5, 122) = 48.1, *p* < 0.001), *R*^*2*^ = 0.663, adj. *R*^*2*^ = 0.649

The model suggests that the significant predictors that directly affect quality of life are anxiety, poor sleep quality, and depression. Cognitive impairment and alexithymia were not found to be significant predictors in the model.

## Discussion

The aim of our study was to map the cognitive and affective characteristics, quality of life, and psychological factors affecting quality of life in patients with unruptured intracranial aneurysm (UIA) and subarachnoid haemorrhage due to rupture of the aneurysm (aSAH). Our results showed that the two groups of patients did not differ significantly from each other in terms of cognitive performance, anxiety, depression, alexithymia, and sleep quality, nor did we find significant differences in how they rated their quality of life. This confirms the fact that the psychological effects of knowledge about UIA can precede physical symptoms and aSAH [11] and that deterioration in quality of life due to psychological burdens such as fear of rupture or depression may occur before bleeding [10]. 34% of the patients showed mild cognitive impairment, 3% dementia, which fits with international estimates for patients with aSAH [17].

We found no significant difference between the two patient groups in terms of their cognitive test results, however the MoCA score of the patients was significantly lower compared to the national normative score, which indicates deterioration in cognitive functioning. Although the overall mean score (24.2 points) did not differ significantly from the clinical cut-off score (24 points) established in the Hungarian validation, the original version of the MoCA score below 26 points suggests a mild cognitive impairment [18], which highlights the cognitive vulnerability of the population of patients. Cognitive deficits may arise from significant affective consequences. The patient population also differed in terms of depression scores compared to healthy controls, who had a much lower average depression score. The overall average of patients fell into the mild depression zone based on the BDI-SF questionnaire domains, however, the study also selected moderately and severely depressed individuals from UIA and aSAH patients, which often required immediate intervention. This confirms the increased prevalence of mood disorders in patients with aneurysms, whether bleeding has occurred or not, which is supported by previous international studies [11, 13].

In general, the patient group had higher mean scores in physical, social, and environmental domains than the much older mean age control sample, but it is surprising and clinically noteworthy that the psychological well-being index is significantly lower, supporting the longer-term psychological consequences of aneurysmal disease. Our correlational analyses also showed a moderate-strong association between anxiety, depression, alexithymia, sleep quality, and perceptions of quality of life. Based on the multivariate linear regression model, three of these variables were found to be independent predictors of quality of life: anxiety, poor sleep quality, and depression. Interestingly, cognitive performance and alexithymia, although correlated with quality of life, did not contribute significantly to the explanatory power of the model. This finding suggests that perceptions of subjective well-being are more dependent on affective and sleep-related variables than on cognitive ability or difficulty in recognizing emotion, which is consistent with the results of previous studies that have examined the UIA and aSAH groups separately [15, 16, 23].

One of the strengths of our study is the heterogeneous sample: we could not find any previous research comparing patients with aSAH, UIA and healthy controls along multiple dimensions. The size of our sample is considered satisfactory, despite the fact that it is a sensitive and difficult to access group of patients, and there were no missing data. An additional strength is that we used validated measures and not only examined differences between groups but also modelled the impact of psychological factors on quality of life. However, there are limitations, such as the cross-sectional nature of the study, which makes it difficult to identify causal relationships, and the fact that, due to the limited number of national studies and the lack of our own healthy control group, we were unable to precisely match age and other demographic variables when comparing with control samples, which may affect the generalizability of the results. The use of self-completion questionnaires has the potential to bias the accuracy of the data. A further limitation is that we did not take into account the location of the aneurysm and the effects of treatment type on cognitive and psychological well-being.

In conclusion, to achieve the highest possible level of recovery and well-being, a holistic approach to the treatment of patients with UIA and aSAH is essential, taking into account not only physical symptoms but also psychological well-being, thus improving the quality of life of patients. The average age of our study sample is low and consists of patients of working age, furthermore, all aSAH patients studied recovered with good medical outcomes, with a modified Rankin Scale rating of 0 or 1, yet live with a variety of psychological symptoms. The worse functional outcome for middle-aged people, such as the impossibility or difficulty of returning to work, causes significant distress for patients, which may also contribute to their reduced cognitive and psychological functioning. The fact that 42% of SAH patients dropped out of the labour market or saw their income decrease may contribute to the decline in quality of life. In our institution, based on the results of the previous pilot study, we started a post-SAH psychological clinic to which we channelled patients who were cognitively or affectively screened [4]. Based on the results of the present study, we have begun to pay special attention to patients with UIA in addition to patients with aSAH. Particularly given that aneurysms are more common among patients with low socio-economic status (SES), which may be related to comorbidities, their complications, and cardiovascular risk factors (e.g., smoking). Patients with low SES definitely receive less attention, even though they are the ones who should receive the most attention, as they do not have the privilege of being able to take proper care of their health and psychological well-being. This underscores the relevance of our research: it is important that intracranial aneurysms management is carried out in an interdisciplinary framework even before bleeding, with psychologists as active members of the team alongside doctors and nurses. It is of particular importance to educate patients and their relatives about the symptoms that may be present and to sensitise the treating staff to the "non-visible" symptoms. Future research should compare in more detail the symptoms of patients with UIA and aSAH, even in different cognitive domains, and should also include studies to detect causality with greater certainty. It would also be worthwhile to compare the patients sample with a healthy sample of working-age in terms of quality of life, which was not possible in this study. Furthermore, we consider it important to examine the cognitive and psychological effects of different aneurysm treatment procedures and the location of the aneurysm, which we plan to do in a future study.

## Data Availability

Our data is not publicly available in accordance with the data protection act. For more information, please contact us by email.

## Abbreviations

AIS: Athens Insomnia Scale
aSAH: Aneurysmal Subarachnoid hemorrhage
BDI-SF: Beck Depression Inventory – Short Form
DM: Diabetes Mellitus
HT: Hypertonia
MCI: Mild Cognitive Impairment
MoCA: Montreal Cognitive Assessment
mRS: Modified Rankin Scale
SES: Socio-economic status
STAI-S/T: State/Trait Anxiety Inventory – State/ Trait Subscales
TAS-20: Toronto Alexithymia Scale – 20 Items
UIA: Unruptured Intracranial Aneurysm
WHOQOL-BREF: World Health Organization Quality of Life Questionnaire – BREF version
